# Liquid Chromatograph-Mass Spectrometry-Based Non-targeted Metabolomics Discovery of Potential Endogenous Biomarkers Associated With Prostatitis Rats to Reveal the Effects of Magnoflorine

**DOI:** 10.3389/fphar.2021.741378

**Published:** 2021-11-01

**Authors:** Yin Yuan, Fei-Xue Dong, Xu Liu, Hong-Bin Xiao, Zhong-Guang Zhou

**Affiliations:** ^1^ Department of Basic Medicine, Heilongjiang University of Chinese Medicine, Harbin, China; ^2^ First Affiliated Hospital of Heilongjiang University of Chinese Medicine, Harbin, China; ^3^ Department of Basic Medicine, College of Pharmacy, Heilongjiang University of Chinese Medicine, Harbin, China; ^4^ Research Institute of Traditional Chinese Medicine, Heilongjiang University of Chinese Medicine, Harbin, China

**Keywords:** metabolomics, UPLC-MS, metabolites, pathways, metabolite biomarkers

## Abstract

Magnoflorine (Mag) has multiple pharmacological activities for the prevention and treatment of prostatitis. However, its molecular mechanisms andpharmacological targets are not clear. In this study, the ultra-performance liquid tandem mass spectrometry-based metabolomics method was used to clarify the intervention of Mag against prostatitis and the biological mechanism. A total of 25 biomarkers associated with the prostatitis model were identified by metabolomics, and a number of metabolic pathways closely related to the model were obtained by MetPA analysis. After given Mag treatment, the results of each indicator were shown that Mag alkaloid could inhibit the development of prostatitis effectively. We found that Mag had regulative effects on potential biomarkers of prostatitis model, which can regulate them to the control group. Our results indicated that alkaloids have an effective intervention therapy for prostatitis, and five types of metabolic pathways closely related to prostatitis model were obtained, including phenylalanine, tyrosine and tryptophan biosynthesis, phenylalanine metabolism, tyrosine metabolism, arginine and proline metabolism, glycine, serine and threonine metabolism, alanine, aspartate and glutamate metabolism. This study has provided the basic experimental data for the development of Mag in the prevention and treatment of prostatitis.

## Introduction

Metabolomics focuses on the comprehensive analysis of overall metabolites in biological samples, including known and unknown metabolites (Zhang et al., 2012; Liang et al., 2015; Li et al., 2018), and give more emphasis on the identification of unknown metabolites. Currently, metabolomics is often analyzed by mass spectrometry (MS) technology in the mode of multiple reaction monitoring (MRM) and parallel reaction monitor (Zhang et al., 2013a; Liang et al., 2016a; Nan et al., 2016). MS plays a dominant analytical platform in metabolomics for its quantitatively and qualitatively measuring metabolites with robustness and high-throughput properties (Zhang et al., 2015; Wang et al., 2019; Pareek et al., 2020). Liquid chromatography-mass spectrometry (LC-MS) is the most widely used metabolomics analysis platform because of its high resolution and sensitivity (Zhang et al., 2013b; Liang et al., 2016b; Zhang et al., 2018). The introduction of ultra-high performance LC improved the efficiency of the chromatographic column, peak resolution and sensitivity. In addition, with a variety of ion sources, UPLC can be associated with a variety of MS detectors, resulting in a broader metabolite coverage (Liang et al., 2017; Qiu et al., 2017; Zhang et al., 2019a). Other MS technologies, such as MS imaging, direct injection MS analysis, etc., can also be used for metabolomics analysis, which provides a variety and broad platforms for metabolomics research (Wang et al., 2016; Zhang et al., 2016; Ren et al., 2018). Metabolomics is a powerful approach to discovering the disease biomarkers, metabolic mechanisms and targets for drugs (Li et al., 2017; Xiong et al., 2020; Xiong et al., 2021).

Prostatitis is a common and multiple disease in middle-aged men, which inevitably affect physical and psychological health of the patients, however, the therapeutic drugs are still rare (Meng et al., 2018). Magnoflorine (Mag), a quaternary aporphine alkaloid, possesses a wide spectrum of pharmacological activities, such as anti-inflammatory, anti-diabetic, immunomodulatory, hypotensive, neuropsychopharmacological, antioxidant, and antifungal activities (Xu et al., 2020). Recently, magnoflorine has received attention due to its multiple pharmacological activities. It has a treatment of diseases including allergies, hypertension, inflammatory ones, osteoporosis, bacterial, fungal infections, and cancer, diabetes, obesity, dementia, or depression (Okon et al., 2020). Mag could prevent inflammatory osteolysis by suppressing MAPK and NF-κB signaling (Sun et al., 2020a). Mag can exert significant anti-inflammatory effects by Inducing Autophagy, Apoptosis and Cell Cycle Arrest (Sun et al., 2020b). Mag could protect against acute lung injury by inhibiting TLR4-mediated NF-κB and MAPK signaling pathways (Guo et al., 2018).

These researches have fully demonstrated the scientific value of Mag in treating prostatic disease. However, it is urgently required togain a better understanding of the molecular mechanisms and targets. We demonstrated the potential of meabolomics approaches to identify candidate biomarker of interest. However, the related material basis is still no sign of a breakthrough. Therefore, we selected the prostatitis model to explore the intervention effect of Mag from the perspective of metabolomics, and provide reliable experimental data for therapeutic material basis of Mag treating prostatitis.

## Materials and Methods

### Chemicals and Materials

Organic reagent of UPLC were purchased from Dikma (United States), the water was obtained from Watsons (China), Sodium pentobarbital (Shanghai chemical, China), Leucine-enkephalin (Sigma, United States), capsaicine (Aladdin industries, United States), Tween 80 (Bodi chemical, China), formamide (kermel, China), and Evans blue (Lanji technology, China) was produced from Shiyitang of Harbin Pharmaceutical Group; Mag (batch number, 201912-183497) has a purity of more than 95% and was purchased from Nanjing Lang Ze Pharmaceutical Technology Co., Ltd. (Nanjing, China) and HPLC chromatograph of Mag was shown in Supplementary Figure S1.

### Animals

Male Wistar rats (10-weeks-old), weighing 260 ± 10 g, were provided by GAP of Heilongjiang University of Traditional Chinese Medicine. Raising temperature at 22 ± 2°C, humidity in 60 ± 5%, the light and dark by turns per 12 h and with natural drink and food. Before the experiment proper starts, feeding a week for animals to be adapted to the surroundings.

### Prostatitis Rat Models

All rats were randomized into control group, model group, and treatment group, each group with 10 rats. Before modeling of prostatitis (Nickel et al., 1991; Rifaioglu et al., 2013), they were given intragastric administration of Magdose (10 mg/ml) for 1 week. After anesthesia, a small incision was taken in the middle-lower abdomen to expose the bladder adequately. 0.1 ml capsaicin solution (1,000 μmol/L) was injected into each prostate gland of the two lobes and double seam in the end. Control group was sham-operated group, no solution was injected after abdominal incision.

### Preparation and Collection of Samples

#### Sample Collection for Evaluating Prostate Protein Exudation

The rats were chosen from each group randomly, and anesthetized with 2% pentobarbital sodium (30 mg/kg). Evans blue solution was injected into the femoral vein at a dose of 50 mg/kg. Half an hour later, the prostates were taken out and weighed, precision addition of 3 ml formamide and rest at room temperature. Centrifugation of the prostatic solution for 15 min at a speed of 12,000 rpm/min. The absorbance value of the supernatant was determined at 620 nm by ultraviolet spectrophotometer.

#### Collection and Pretreatment of Blood Samples

Acquisition of rat blood via the abdominal aorta, and blood samples were centrifuged 15 min at a speed of 5,000 rpm/min, to obtain the plasma samples, and frozen at −80°C. The blood sample had to be thawed before analysis, and the protein was precipitated by methanol and ethanol (1:1). The volume ratio of organic solvent to the blood sample was 4:1. Shock for 15 min, the samples were centrifuged 10 min at a speed of 13,000 rpm/min. The supernatant was filtered through 0.22 µm filter membrane for analysis.

### UPLC-MS Instruments and Experimental Conditions

The samples were analyzed by ACQUITY UPLC T3 (100 mm × 2.1 mm i. d., 1.8μm, Waters, United States) at 40°C with a flow rate of 0.4 ml/min. Using 0.1% formic acid-acetonitrile (A) and 0.1% formic acid-water (B) as the mobile phase. The elution gradient: 0–3 min, 1–55% A; 3∼9 min, 55–90% A; 9–12 min, 90–99% A. The G2Si-MS/MS (Waters Q-TOFSYNAPT™, Waters Corp, Milford, United States) equipped with EIS was used for analysis. The cone bore voltage in both positive and negative ion modes were 30 V. Capillary voltage was 3.0 kV, temperature of desolvation was 400°C, and ion source was 110°C. Desolvent and cone gas flow velocity were 600 L/h and 60 L/h, respectively. Data were captured under centroid mode and the range of mass scan was 50–1,000 Da. For precise mass detection, leucine-enkephalin [(M + H)^+^ = 556.2771 and (M–H)^-^ = 554.2615] was selected as the lock-mass solution tothe concentration of 0.2 ng/ml.

### Multivariate Data Analyses

Metabolome data was analysed by software processing system of Waters Progenesis® QI. Unsupervised principal component analysis (PCA) and supervised orthogonal partial least squares analysis (OPLS-DA) were performed by software of EZinfo to obtain the metabolic profiling and score plot map. SPSS V22.0 was used for each group above data analysis. One-way ANOVA method and Student’s t-test were used for statistical analysis. Ions with VIP>1 and *p* < 0.05 in *t*-test were chosen as potential biomarkers associated with prostatitis model. The components were analyzed through multiple databases, such as chemspider, HMDB, Metaboanalyst and KEGG pathway.

## Results

### Behavioral Assessment of Prostatitis

Behavioral changes of the rats were recorded every 10 min within half an hour after awakening, including eye closure and activity status. The results showed that there was a very significant discrepancy between the control group and the model group (*p* < 0.01), which indicating that after injected capsaicin into prostatic tissue, rats in the model group developed pain symptoms, as showed in Supplementary Table S1.

### Prostate Protein Exudation

The content of Evans blue in each group was assessed by the absorbance value measured at 620 nm. The results showed that the protein exudation in the model group was obviously higher than the control group (*p* < 0.01). It means that there were injury and inflammation in rats of model group, as showed in Supplementary Table S2.

### Multivariate Statistical Analysis

**FIGURE 1:**
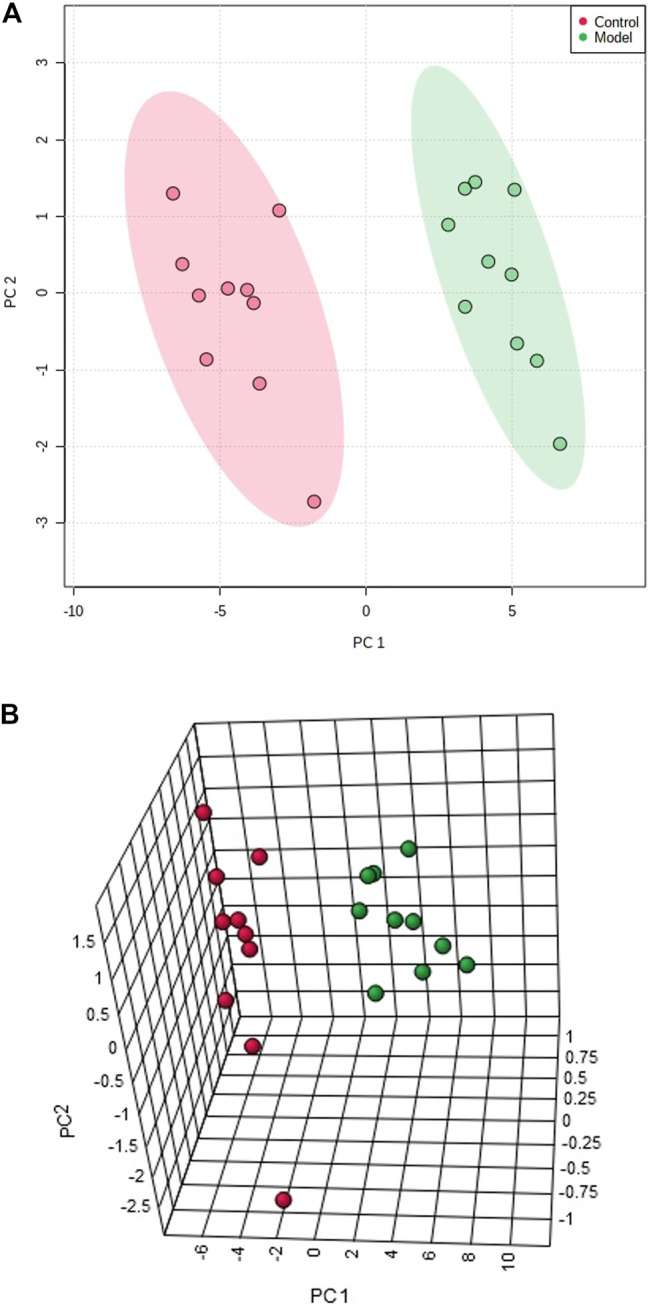
PCA score plots based on lipid profile for discriminating control group and model group using metabolomics method based on liquid chromatography coupled with mass spectrometry **(A)**. PCA 3-D score plot based on lipid metabolites discriminating control and model groups using metabolomics method based on liquid chromatography coupled with mass spectrometry **(B)**.

**FIGURE 2:**
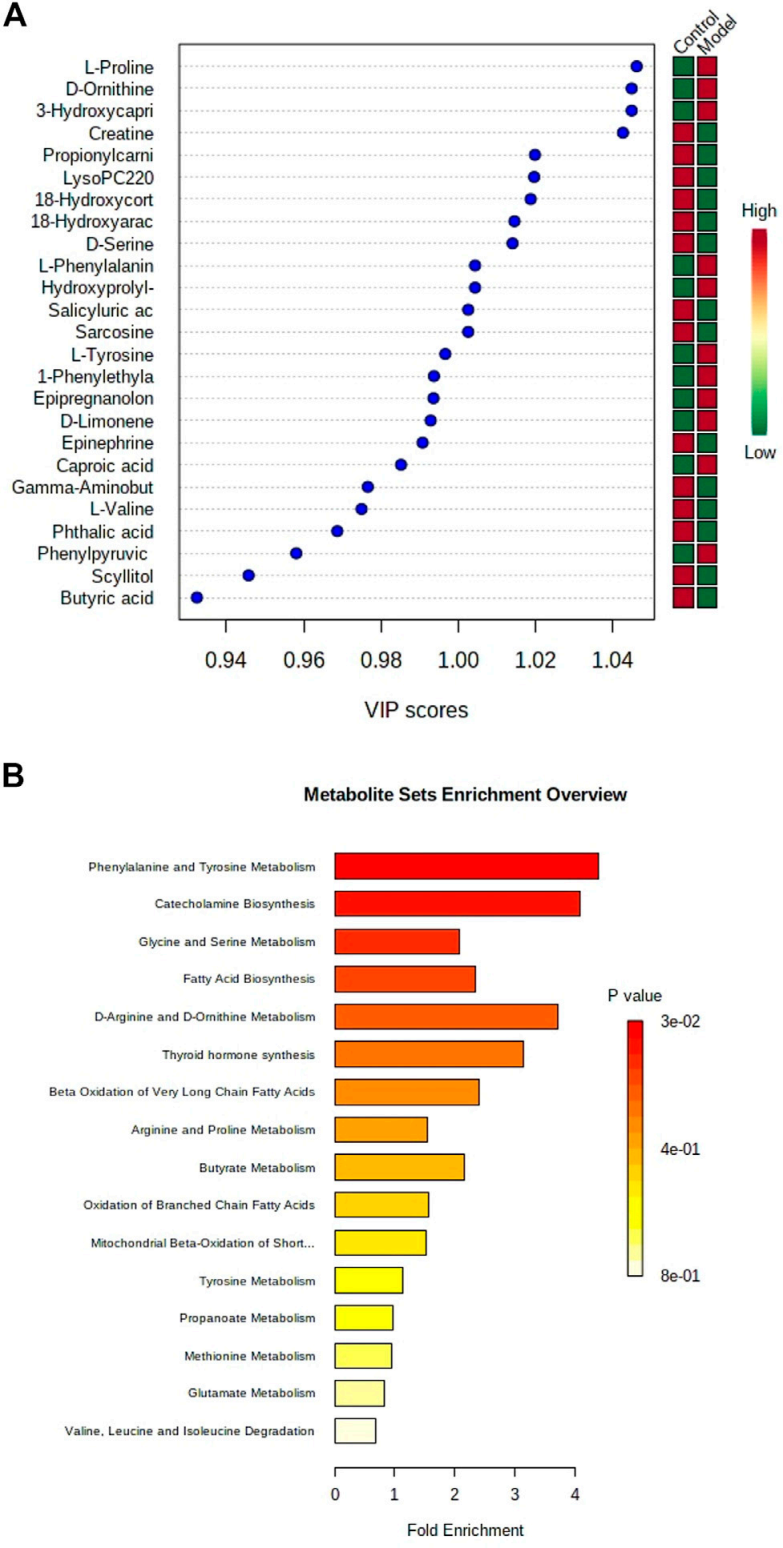
VIP plot of lipid biomarker candidates scanned by OPLS-DA analysis using metabolomics method based on liquid chromatography coupled with mass spectrometry **(A)**. Enrichment analysis of potential lipid metabolites in control group and model group using metabolomics method based on liquid chromatography coupled with mass spectrometry **(B)**.

The multivariate pattern recognition was utilized to analyze the blood data, to observe whether the preparation of prostatitis model resulted in changes in endogenous components of the body. PCA and 3-D score plots of the control group and the model group are shown in Figures 1A,B. It can be observed inthe results that the two groups can be distinguished clearly, which suggesting that the endogenous metabolites of the body have been disturbed significantly after injection of capsaicin. The VIP-plot (Figure 2A) shows that the farther the ion is from the original center, the greater its contribution to the fractionation. At the same time, thefundamentalmetabolic products were identified by combining the VIP value and t-test results of inter-group.

### Identification of Biomarkers

According to measuring accurate molecular mass of each ion, and the fragment information of MS/MS was used to analyze the pyrolysis mode, potential biomarkers were retrieved and matched through by multiple databases and software, as well as combined with the analysis of literature to identify the possible chemical structure of the potential biomarkers. Using the identification method, we finally identified 25 biomarkers in positive and negative ion modes, and the detailed information is shown in the Supplementary Table S3. The changes of these biomarkers in the metabolism of the body, indicated that the capsaicin-induced prostatitis can disturb normal metabolism and alter metabolic expression. The metabolic pathways associated with phenylalanine and tyrosine metabolism, catecholamine biosynthesis, glycine and serine metabolism, fatty acid biosynthesis, D-arginine and D-ornithine metabolism, and thyroid hormone synthesis were presented in Figure 2B and Supplementary Table S4.

### Intervention Effects of Mag

From the results of eye movement group and activation group (Supplementary Table S5), we can see that contrasting with the control group, the model group and treatment group had a very significant difference (*p* < 0.01). It concludes that Mag can effectively inhibit the development of prostatitis. As showed in Supplementary Table S6, contrasting with the control group, the model group had a very significant difference (*p* < 0.01); contrasting with the model group, treatment group had a very significant difference (*p* < 0.01). The results showed that Mag could dramatically improve the inflammation.

### Regulation of Biomarker and Metabolic Pathway

**FIGURE 3:**
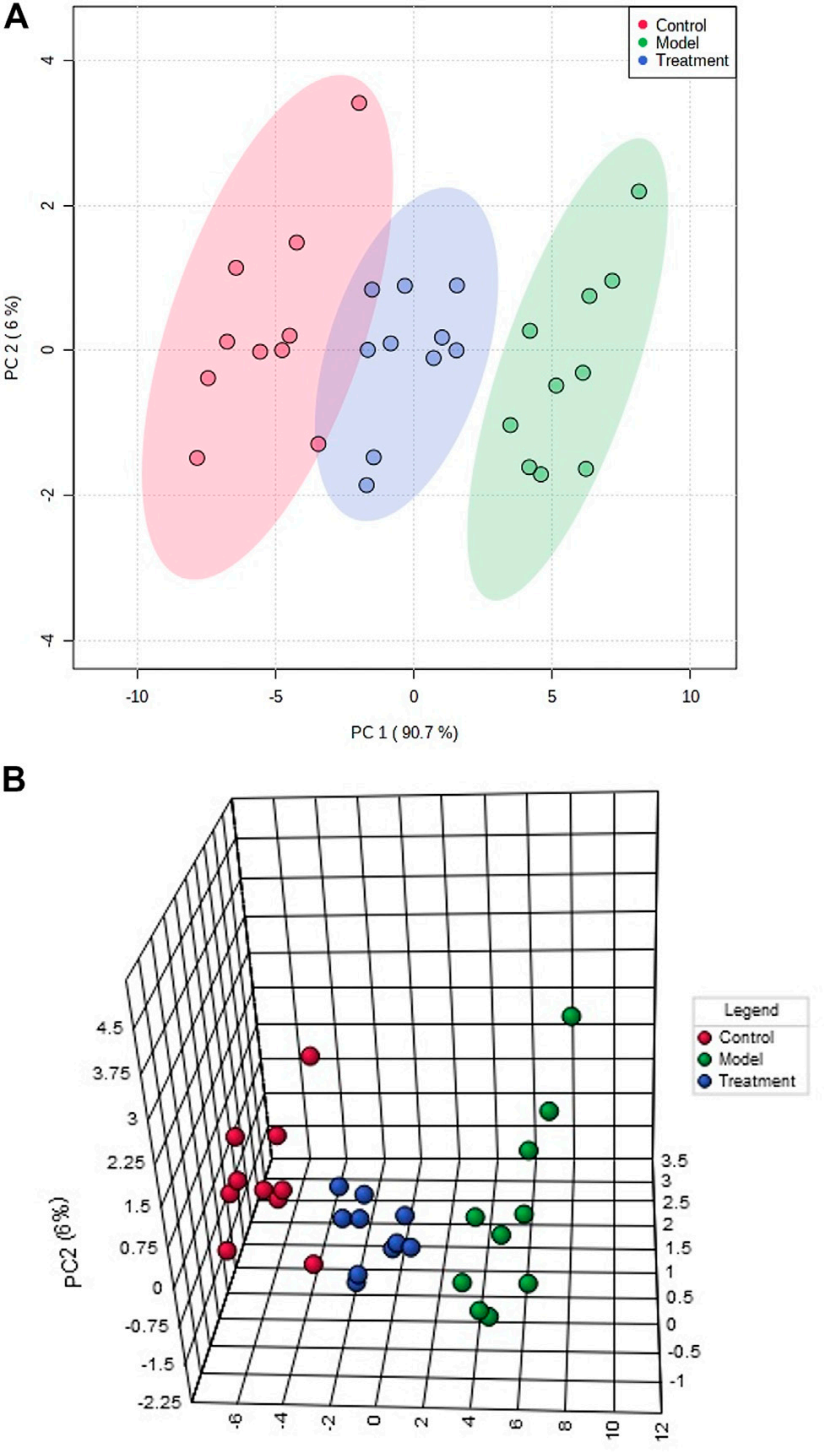
The score plot of control group, model group and treatment groups in the PCA model using metabolomics method based on liquid chromatography coupled with mass spectrometry **(A)**. PCA 3-D score plot of control group, model group and treatment groups in the PCA model using metabolomics method based on liquid chromatography coupled with mass spectrometry **(B)**.

Depending on the analytical methods mentioned above, the changes of endogenous metabolites in each group were observed, to compare the intervention effect of each group on the metabolites of prostatitis model. PCA and 3-D score plots (Figures 3A,C) showed that the samples in each group were clustered and separated between groups. The control group could be clearly distinguished from the model group, suggesting that the endogenous metabolites of the body were disturbed obviously after modeling. Meanwhile, it was found that the metabolic profile of the treatment group was closer to the control group, which indicated that the application of Mag has been acted positively to intervene the prostatitis rats.

**FIGURE 4:**
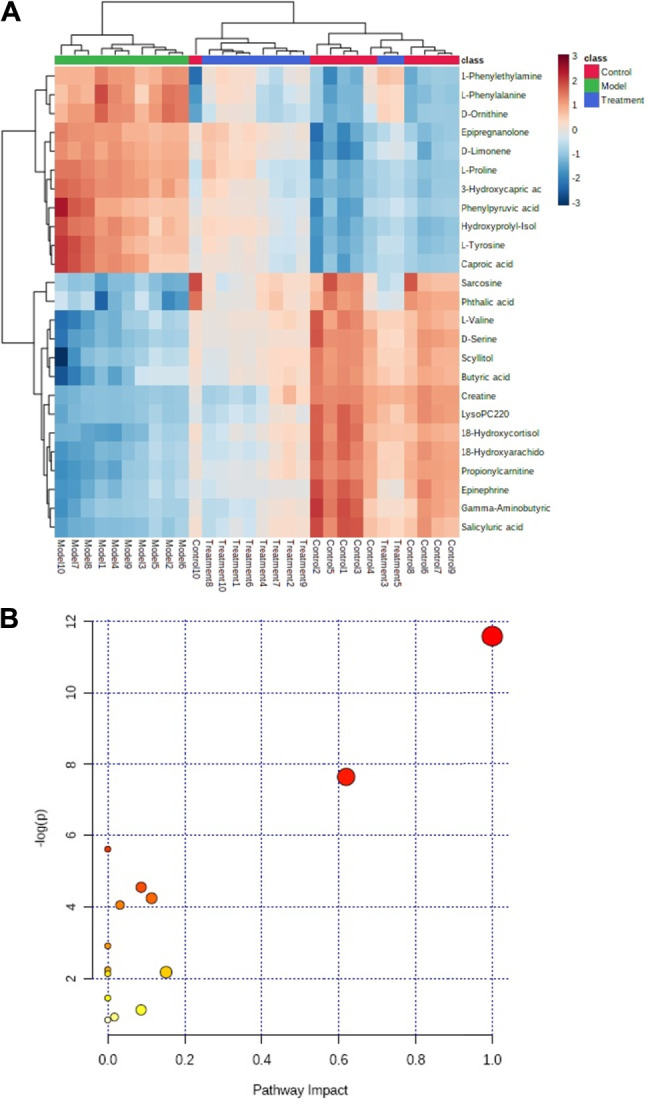
Heatmap of potential metabolites in control group, model group and treatment groups **(A)**. Regulatory pathway analysis of magnoflorine treatment **(B)**.

Based on the 25 biomarkers obtained from the group of control and model, to analyze the variation tendency of these potential biomarkers in prostatitis model rats after oral administration of Mag. We found that the application of Mag could regulate the content of potential biomarkers of prostatitis rat model to the control group (Figure 4A). The metabolic pathways associated with the phenylalanine, tyrosine and tryptophan biosynthesis, phenylalanine metabolism, tyrosine metabolism, arginine and proline metabolism, glycine, serine and threonine metabolism, alanine, aspartate and glutamate metabolism for the intervention effects of Mag were presented in Figure 4B.

## Discussion

In this study, capsaicin was used to prepare the rat model of prostatitis. We evaluated the prostatitis model by classical behavior and prostate protein exudation. The results of eye movement and activity, protein exudation and histopathological sections showed that the pain symptoms of prostatitis occurred after capsaicin injection into the prostatic tissue of the rats, which indicated that the rat model of prostatitis was replicated successfully. According to the relevant consequences, it can be seen that the Mag alkaloid could significantly improve the symptoms of prostatitis. The difference of metabolic profiles of endogenous molecules *in vivo* can bee analyzed by means of metabolomics method (Wang et al., 2013; Wang et al., 2014; Zhang et al., 2019b). They were grouped into two clusters distinctly, means that the replication of the model was successful at the metabolomics level. A total of 25 biomarkers related to the rat model of prostatitis were identified in positive and negative modes, and the metabolic pathways associated with the prostatitis model were discovered.

Based on analysis of contour characterization and pattern recognition, it was found that oral administration of Mag alkaloids could affect the potential biomarkers of the prostatitis model, and the content of them tends to be regulated to near the level of the control group. Among 18 potential biomarkers of prostatitis rat model, 11 potential biomarkers related to the prostatitis model were regulated, which may improve the situation of endogenous metabolites. Levels of 18-hydroxycortisol, 18-hydroxyarachidonic acid, uric acid and3-hydroxycapric acid were adjusted greatly. Uric acid has antioxidant property that prevents the development of inflammatory environments (Zhang et al., 2014b; Qiu et al., 2020; Zhang et al., 2020). The remarkable reversal of L-leucine and L-tyrosine may correct the abnormality of amino acid metabolism (Zhang et al., 2014a; Song et al., 2017; Zhang et al., 2017). Regulation of LysoPC(22:0) has an important effect on lipid metabolism. The above results indicated that alkaloids have an effective intervention therapy for prostatitis, and five types of metabolic pathways closely related to prostatitis model were obtained, including phenylalanine, tyrosine and tryptophan biosynthesis, phenylalanine metabolism, tyrosine metabolism, arginine and proline metabolism, glycine, serine and threonine metabolism, alanine, aspartate and glutamate metabolism.

Magnoflorine (Mag) has multiple pharmacological activities for the prevention and treatment of prostatitis. The promising metabolomics strategy has been used for reveling the metabolic biomarkers, ans efficacy mechanisms, as well as pharmacological targets for drugs (Qiu et al., 2021; Ren et al., 2021; Zhang et al., 2021). In this work, metabolomics method was used to clarify the intervention of Mag against prostatitis and the biological mechanism. The biomarkers associated with the prostatitis model were identified and a number of metabolic pathways closely related to the model were also obtained. It was found that Mag had regulative effects on potential biomarkers, which can regulate them to the control group and five types of metabolic pathways closely related to prostatitis model were obtained, including phenylalanine, tyrosine and tryptophan biosynthesis, phenylalanine metabolism, tyrosine metabolism, etc. This work has provided the experimental data for the development of Mag in the treatment of prostatitis.

## Conclusion

This work used UPLC-MS/MS to explore the blood metabolomics and therapeutic effects of Mag against chronic non-bacterial prostatitis. Through the analysis of the metabolic profiles, there were 25 biomarkers have been defined, which were closely relevant to the occurrence and development of prostatitis. The results showed that Mag could regulate the expression abnormality of biomarkers, and adjust the abnormal metabolic profile close to the normal condition. Mag could inhibit the development of inflammatory symptom, and effectively intervene the treatment of prostatitis. It serves as a theoretical basis for further study of the therapeutic and preventive effects of Mag on chronic non-bacterial prostatitis.

## Data Availability

The original contributions presented in the study are included in the article/Supplementary Material, further inquiries can be directed to the corresponding authors.
