# Corrigendum: Liquid chromatograph-mass spectrometry-based non-targeted metabolomics discovery of potential endogenous biomarkers associated with prostatitis rats to reveal the effects of magnoflorine

**DOI:** 10.3389/fphar.2022.1034753

**Published:** 2022-10-12

**Authors:** Yin Yuan, Fei-Xue Dong, Xu Liu, Hong-Bin Xiao, Zhong-Guang Zhou

**Affiliations:** ^1^ Department of Basic Medicine, Heilongjiang University of Chinese Medicine, Harbin, China; ^2^ First Affiliated Hospital of Heilongjiang University of Chinese Medicine, Harbin, China; ^3^ Department of Basic Medicine, College of Pharmacy, Heilongjiang University of Chinese Medicine, Harbin, China; ^4^ Research Institute of Traditional Chinese Medicine, Heilongjiang University of Chinese Medicine, Harbin, China

**Keywords:** metabolomics, UPLC-MS, metabolites, pathways, metabolite biomarkers

In the published article, there was an error in Supplementary Tables S1, S2, S5, S6. We have submitted the wrong version of the supplementary material. The correct material appears in Supplementary Material.

The authors apologize for this error and state that this does not change the scientific conclusions of the article in any way. The original article has been updated.

