# Supplementary material for: Liquid Chromatograph-Mass Spectrometry-Based Non-targeted Metabolomics Discovery of Potential Endogenous Biomarkers Associated With Prostatitis Rats to Reveal the Effects of Magnoflorine
Source: Front Pharmacol. 2021 Nov 1;12:741378. doi: 10.3389/fphar.2021.741378 (PMC8591080; doi:10.3389/fphar.2021.741378)
Supplement: Supplementary file 1 [file Table1.docx]

TableS1 The behavioral comparison results (Mean±SD)

| Group | Eye movement | Activity |  |
| --- | --- | --- | --- |
| Control | 1.37±0.56 | 1.5±0.57 |  |
| Model | 4.27±0.69** | 4.5±0.63** |  |

**Compared with the blank group, there were significant differences, p<0.01

TableS2 Plasma protein exudation (Mean±SD)

| Group | Prostate protein exudation |
| --- | --- |
| Control | 20.31±1.00 |
| Model | 29.17±0.80** |

**Compared with the blank group, there were significant differences, p<0.01

TableS3 The identification information of the biomarkers related to rat model.

| No | Rt | m/z | Compound ID | Adducts | Formula | ppm | Compound |
| --- | --- | --- | --- | --- | --- | --- | --- |
| 1 | 0.91 | 118.0869 | HMDB00883 | M+H | C5H11NO2 | 0.79 | L-Valine |
| 2 | 4.93 | 106.0505 | HMDB03406 | M+H | C3H7NO3 | 0.80 | D-Serine |
| 3 | 0.64 | 132.0776 | HMDB00064 | M+H | C4H9N3O2 | 1.94 | Creatine |
| 4 | 2.15 | 104.0711 | HMDB00112 | M+H | C4H9NO2 | -0.90 | Gamma-Aminobutyric acid |
| 5 | 1.21 | 184.0981 | HMDB00068 | M+H | C9H13NO3 | 1.26 | Epinephrine |
| 6 | 0.93 | 182.0825 | HMDB00158 | M+H | C9H11NO3 | 1.02 | L-Tyrosine |
| 7 | 1.19 | 166.0872 | HMDB00159 | M+H | C9H11NO2 | 0.21 | L-Phenylalanine |
| 8 | 2.63 | 117.0916 | HMDB00535 | M+H | C6H12O2 | 0.09 | Caproic acid |
| 9 | 0.83 | 90.0553 | HMDB00271 | M+H | C3H7NO2 | -2.13 | Sarcosine |
| 10 | 7.95 | 167.0349 | HMDB02107 | M+H | C8H6O4 | 1.85 | Phthalic acid |
| 11 | 1.33 | 122.0971 | HMDB02017 | M+H | C8H11N | 1.28 | 1-Phenylethylamine |
| 12 | 6.31 | 401.1922 | HMDB00418 | M+Na | C21H30O6 | -1.83 | 18-Hydroxycortisol |
| 13 | 5.88 | 343.2262 | HMDB06245 | M+Na | C20H32O3 | 0.91 | 18-Hydroxyarachidonic acid |
| 14 | 7.86 | 580.4370 | HMDB10398 | M+H | C30H62NO7P | 1.83 | LysoPC(22:0) |
| 15 | 7.16 | 341.2469 | HMDB01471 | M+Na | C21H34O2 | 1.03 | Epipregnanolone |
| 16 | 0.72 | 203.0535 | HMDB06088 | M+Na | C6H12O6 | 0.16 | Scyllitol |
| 17 | 3.59 | 243.1326 | HMDB28866 | M-H | C11H20N2O4 | -0.95 | Hydroxyprolyl-Isoleucine |
| 18 | 0.87 | 114.0715 | HMDB00162 | M-H | C5H7NO2 | 1.70 | L-Proline |
| 19 | 1.46 | 87.0601 | HMDB00039 | M-H | C4H6O2 | -2.21 | Butyric acid |
| 20 | 6.38 | 187.1320 | HMDB02203 | M-H | C10H20O3 | -1.33 | 3-Hydroxycapric acid |
| 21 | 5.46 | 131.0976 | HMDB03374 | M-H | C5H10N2O2 | -0.61 | D-Ornithine |
| 22 | 2.79 | 135.1328 | HMDB03375 | M-H | C10H14 | -1.62 | D-Limonene |
| 23 | 3.48 | 194.0437 | HMDB00840 | M-H | C9H9NO4 | -0.94 | Salicyluric acid |
| 24 | 1.08 | 216.1401 | HMDB00824 | M-H | C10H17NO4 | 1.05 | Propionylcarnitine |
| 25 | 0.97 | 163.0555 | HMDB00205 | M-H | C9H6O3 | 1.15 | Phenylpyruvic acid |

Table. S4 The differential metabolite enrichment analysis

|  | total | expected | hits | Raw p |
| --- | --- | --- | --- | --- |
| Phenylalanine and Tyrosine Metabolism | 28 | 0.684 | 3 | 0.03 |
| Catecholamine Biosynthesis | 20 | 0.488 | 2 | 0.08 |
| Glycine and Serine Metabolism | 59 | 1.44 | 3 | 0.17 |
| Fatty Acid Biosynthesis | 35 | 0.854 | 2 | 0.21 |
| D-Arginine and D-Ornithine Metabolism | 11 | 0.269 | 1 | 0.24 |
| Thyroid hormone synthesis | 13 | 0.317 | 1 | 0.28 |
| Beta Oxidation of Very Long Chain Fatty Acids | 17 | 0.415 | 1 | 0.35 |
| Arginine and Proline Metabolism | 53 | 1.29 | 2 | 0.38 |
| Butyrate Metabolism | 19 | 0.464 | 1 | 0.38 |
| Oxidation of Branched Chain Fatty Acids | 26 | 0.635 | 1 | 0.48 |
| Mitochondrial Beta-Oxidation of Short Chain Saturated Fatty Acids | 27 | 0.659 | 1 | 0.49 |
| Tyrosine Metabolism | 72 | 1.76 | 2 | 0.54 |
| Propanoate Metabolism | 42 | 1.03 | 1 | 0.65 |
| Methionine Metabolism | 43 | 1.05 | 1 | 0.66 |
| Glutamate Metabolism | 49 | 1.2 | 1 | 0.71 |
| Valine, Leucine and Isoleucine Degradation | 60 | 1.46 | 1 | 0.78 |

TableS5 Results of behavioral comparison of each group of rats after modeling(Mean±SD)

| Group | Eye movement | Activity |
| --- | --- | --- |
| Control | 1.37±0.56 | 1.5±0.57 |
| Model | 4.27±0.69** | 4.5±0.63** |
| Treatment | 3.03±0.89## | 2.43±0.9## |

* Compared with the CON group, there was significant difference, p<0.05；** Compared with the CON group, there was very significant difference, p<0.01;

# Compared with the MOD group, there was significant difference, p<0.05；## Compared with the MOD group, there was very significant difference, p<0.01

TableS6 Results of comparison of prostate protein exudation(Mean±SD)

| Group | Prostate protein exudation |
| --- | --- |
| Control | 20.31±1.00 |
| Model | 29.17±0.80** |
| Treatment | 26.02±1.35## |

* Compared with the CON group, there was significant difference, p<0.05；** Compared with the CON group, there was very significant difference, p<0.01; # Compared with the MOD group, there was significant differencep<0.05；## Compared with the MOD group, there was very significant difference, p<0.01

Table.S7 The metabolite pathways analysis

|  | **Total** | **Expected** | **Hits** | **Raw p** | **Impact** |
| --- | --- | --- | --- | --- | --- |
| Phenylalanine, tyrosine and tryptophan biosynthesis | 4 | 0.06 | 3 | 0.00 | 1.00 |
| Phenylalanine metabolism | 12 | 0.17 | 3 | 0.00 | 0.62 |
| Tyrosine metabolism | 42 | 0.59 | 2 | 0.11 | 0.15 |
| Arginine and proline metabolism | 38 | 0.53 | 3 | 0.01 | 0.11 |
| Glycine, serine and threonine metabolism | 34 | 0.47 | 3 | 0.01 | 0.09 |
| Alanine, aspartate and glutamate metabolism | 28 | 0.39 | 1 | 0.33 | 0.09 |
| Butanoate metabolism | 15 | 0.21 | 2 | 0.02 | 0.03 |
| Glycerophospholipid metabolism | 36 | 0.50 | 1 | 0.40 | 0.02 |
| Aminoacyl-tRNA biosynthesis | 48 | 0.67 | 4 | 0.00 | 0 |
| D-Arginine and D-ornithine metabolism | 4 | 0.06 | 1 | 0.05 | 0 |
| Valine, leucine and isoleucine biosynthesis | 8 | 0.11 | 1 | 0.11 | 0 |
| Ubiquinone and other terpenoid-quinone biosynthesis | 9 | 0.13 | 1 | 0.12 | 0 |
| Pantothenate and CoA biosynthesis | 19 | 0.26 | 1 | 0.24 | 0 |
| Valine, leucine and isoleucine degradation | 40 | 0.56 | 1 | 0.43 | 0 |


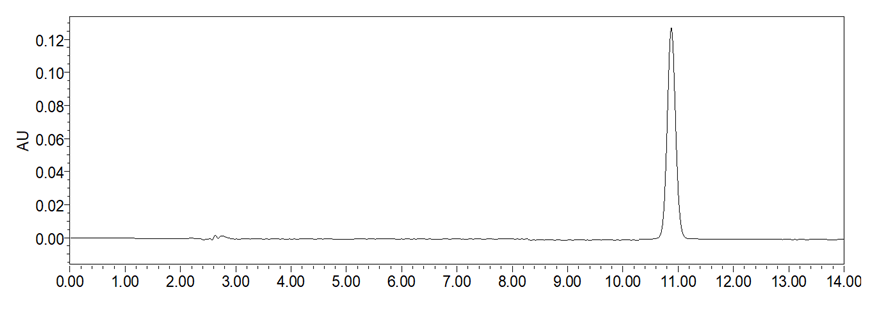


Fig.S1. HPLC chromatograph of *Mag.* A Kromasil-C18 column (4.6 mm×250 mm,5μm), acetonitrile-50 mmol.L-1 potassium dihydrogen phosphate solution (phosphoric acid adjusted pH 3.0) (45:55), containing 12.5 mmol/L sodium dodecyl sulfate as mobile phase, volume flow rate 1 mL/min, column temperature 30℃, detection wavelength 275 nm; injection volume 10 μL。
